# Effect of Therapeutic Inhibition of TNF on Circulating Endothelial Progenitor Cells in Patients with Rheumatoid Arthritis

**DOI:** 10.1155/2013/537539

**Published:** 2013-10-09

**Authors:** F. R. Spinelli, A. Metere, C. Barbati, M. Pierdominici, C. Iannuccelli, B. Lucchino, F. Ciciarello, L. Agati, G. Valesini, M. Di Franco

**Affiliations:** ^1^Department of Internal Medicine and Medical Specialities, Rheumatology Unit, Sapienza Università di Roma, Viale del Policlinico 155, 00161 Roma, Italy; ^2^Section of Biomarkers in Degenerative Diseases, Department of Cell Biology and Neurosciences, Istituto Superiore di Sanità, Viale Regina Elena 299, 00161 Rome, Italy; ^3^Department of Cardiovascular and Respiratory Sciences, Sapienza University of Rome, Viale del Policlinico 155, 00161 Roma, Italy

## Abstract

Endothelial dysfunction has been detected in RA patients and seems to be reversed by control of inflammation. Low circulating endothelial progenitor cells (EPCs) have been described in many conditions associated with increased cardiovascular risk, including RA. The aim of this study was to investigate the effect of inhibition of TNF on EPCs in RA patients. Seventeen patients with moderate-severe RA and 12 sex and age-matched controls were evaluated. Endothelial biomarkers were tested at baseline and after 3 months. EPCs were identified from peripheral blood mononuclear cells by cytofluorimetry using anti-CD34 and anti-vascular endothelial growth factor-receptor 2. Asymmetric dimethylarginine (ADMA) was tested by ELISA and flow-mediated dilatation (FMD) by ultrasonography. Circulating EPCs were significantly lower in RA patients than in controls (*P* = 0.001). After 3 months EPCs increased significantly (*P* = 0.0006) while ADMA levels significantly decreased (*P* = 0.001). An inverse correlation between mean increase in EPCs number and mean decrease of DAS28 after treatment was observed (*r* = −0.56, *P* = 0.04). EPCs inversely correlated with ADMA (*r* = −0.41, *P* = 0.022). No improvement of FMD was detected. Short-term treatment with anti-TNF was able to increase circulating EPCs concurrently with a proportional decrease of disease activity suggesting that therapeutic intervention aimed at suppressing the inflammatory process might positively affect the endothelial function.

## 1. Introduction

Rheumatoid Arthritis (RA), as other autoimmune systemic diseases, is associated with increased cardiovascular morbidity and mortality [1], mostly attributable to accelerated atherosclerotic process [2]. Data in the literature demonstrated that inflammatory nature of RA contributes to the excess of atherosclerosis observed in this disease [3]. Rheumatoid synovia and atherosclerotic plaque share a common inflammatory cellular and molecular *milieu* characterized by an activated endothelial phenotype, expression of the same pattern of adhesion molecules, cytokines, and infiltrating leucocytes [4].

Impairment of endothelial function represents the earliest and reversible stage of atherosclerotic plaque formation, originating from the loss of protective antioxidant and anti-inflammatory systems [5]. Integrity of vascular endothelium is essential for arterial wall functions and homeostasis, and its dysfunction represents the key event which subsequently leads to vascular wall disorders. Less than twenty years ago, Asahara and coll firstly identified endothelial progenitor cells (EPCs) as precursors circulating in peripheral blood, mobilized form bone marrow, and able to differentiate *in situ* into endothelial cells; such cells contribute to the recovery of injured endothelium, thus, limiting atherosclerotic plaque formation [6–8]. Mobilization and differentiation of the EPCs is known to be regulated by nitric oxide (NO) produced through the activation of the endothelial NO synthase (eNOS) [9]. 

The number and functional activity of EPCs seem to influence cardiovascular risk. An inverse correlation between the number of EPCs and the Framingham risk factor score has been demonstrated [7], and defective number and function of these cells have been found in different clinical conditions associated with an increased cardiovascular risk [10].

Endothelial dysfunction has been documented in both long-standing [11] and early RA patients [12, 13] with Doppler ultrasound assessment of brachial artery flow-mediated dilatation (FMD) or evaluation of artery wall stiffness [14]. An improvement of endothelial function after treatment has been demonstrated by several authors [15–19]. Patients with RA also show a reduced number of circulating EPCs, which inversely correlates with disease activity and seems to be responsive to glucocorticoids [20]. Moreover, an association between the endogenous eNOS inhibitor asymmetric dimethyl arginine (ADMA) and the number of circulating EPCs has been detected in RA patients who have no other cardiovascular risk factors [21].

To date, anti-TNF agents represent a milestone of RA treatment. Given the evident role of TNF in atherosclerosis, a beneficial effect of TNF inhibition has been postulated; however, observational studies and data form registries did not always demonstrate a decrease in cardiovascular events [1]. Long-term controlled studies, directly evaluating the effect of this class of drugs on atherosclerotic process progression, are needed.

The aim of our study was to investigate the effect of short-term subcutaneous administration of anti-TNF drugs on EPCs number in patients with active RA.

## 2. Materials and Methods

### 2.1. Patients and Controls

Consecutive patients affected by RA according to 1987 criteria [22], designated to start subcutaneous anti-TNF drugs, were recruited from the “biological drugs-dedicated outpatient clinic” of the Rheumatology Unit of Sapienza University of Rome. All patients were prospectively followedup for at least 3 months. As control, 12 age and sex-matched healthy subjects were studied. All patients signed an informed consent before entering the study. At recruitment, demographic and clinical data, and comorbidities were recorded. Patients and controls were excluded in case of a diagnosis of cardiovascular diseases, chronic kidney failure, dyslipidemia, and/or diabetes. Before starting anti-TNF, patients were screened for latent tuberculosis and hepatitis virus B and C.

### 2.2. Disease Activity Assessment

RA disease activity was evaluated at baseline, and after 3 months of anti-TNF treatment, by 28-joint disease activity score (DAS28).

### 2.3. Blood Samples

Blood samples were collected from each patient at baseline and 3 months later. Heparinized vials were used to test EPCs on the same day of the blood draw. The remaining samples were centrifuged at 3000 ×g for 10 minutes at room temperature and serum collected and frozen at −80°C until analyzed.

As for control group, blood samples from healthy subjects were collected on the same day of baseline patients' visit.

### 2.4. Circulating Endothelial Progenitor Cell Analysis

**Figure 1:**
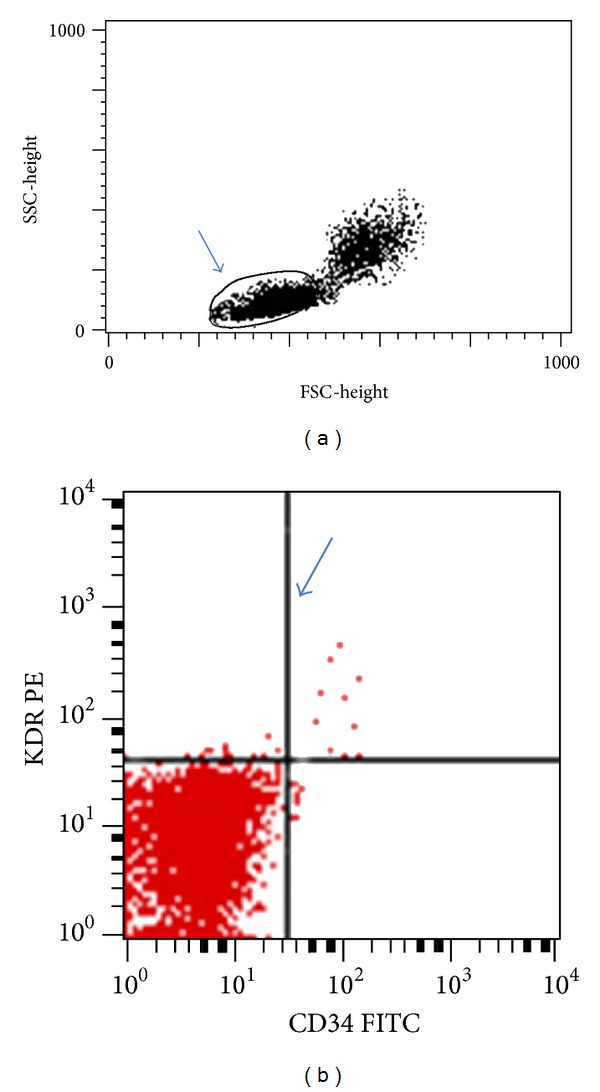
Flow cytometry analysis of CD34+ KDR+ cells. Representative flow cytometry plots obtained from a healthy control is shown. (a) Forward scatter (FSC), and side scatter (SSC) with lymphocytes gate are indicated. (b) Double fluorescence with PE-labeled KDR and FITC-labeled CD34 antibodies. In top right quadrant CD34/KDR double-positive cells are indicated. Quadrants were set on the basis of isotype controls.

Peripheral blood mononuclear cells (PBMCs) were obtained by density gradient centrifugation (Lympholyte-H; Cedarlane Laboratories, Hornby, Ontario, Canada), and phenotypic characterization was performed as previously described by Vasa et al. [23]. In brief, after incubation with FcR-bloking reagent (Miltenyi Biotec, Bergisch-Gladbach, Germany), cells were incubated for 30 min on ice with phycoerythrin (PE)-labeled mAb anti-CD34 (BD Immunocytometry Systems, San Jose, CA) and allophycocyanin (APC)-labeled mAb anti VEGF R2/KDR (R&D Systems, Minneapolis, MN). Appropriate isotype controls were used. Acquisition was performed on a FACS Calibur (BD Immunocytometry Systems) and included 100.000 to 400.000 events per sample. Data were analyzed using the CellQuest Pro software (BD Immunocytometry Systems). EPCs were defined as to CD34/KDR double-positive cells, and their number was expressed as a percentage of cells within the lymphocyte gate [21, 23]. A representative dotplot is shown in Figure 1.

### 2.5. ADMA

ADMA serum levels were detected by a commercial human enzyme linked immunosorbent assay (ELISA) kit (Vinci Biochem, Florence, Italy), according to manufacturer's instructions. Sera were tested in triple and result expressed as mean value ± standard deviation.

### 2.6. Assessment of Flow Mediated Dilatation

Flow mediated dilation in response to reactive hyperemia (endothelium dependent vasodilatation) was evaluated on brachial artery by employing a high-resolution B-mode Doppler (ATL HDI 5000 with a 7.4 MHz linear-array transducer) and following the guidelines published by the International Brachial Arterial Reactivity Task Force [24].

All subjects were evaluated fasting between 8 and 11 AM, in a quiet and stable temperature environment. A straight, nonbranching segment of the brachial artery 5–15 cm above the antecubital fossa was identified by a B-mode longitudinal scan. Vessel diameter was recorded in a segment with clear anterior and posterior intimal interfaces between the lumen and vessel wall at rest and during reactive hyperemia. Brachial artery diameter was measured offline by an automatic edge-detection system. A blood pressure cuff was then inflated around the forearm to a supra-systolic pressure (at least 50 mm Hg above the systolic pressure to occlude arterial inflow) for the standardized length of 5 minutes. Measurement of the maximal diameter of the artery was taken 45 to 60 seconds after cuff release. Absolute FMD was expressed as: (postdeflation diameter-resting diameter); FMD relative values were also calculated as percent change from the baseline diameter as follows: 100% × ((postdeflation diameter − resting diameter)/resting diameter).

Two cardiologists (FC and LA), blinded to participants' clinical data, interpreted the ultrasound results using an offline method. The intra- and interobserver variability of the FMD were calculated within the study population by plotting the patients' FMD estimates from each measurement against the estimates by two independent measurements. The estimate's standard error was calculated using this plot. The intra- and interobserver variability were 4.2% and 5.1%, respectively.

In order to evaluate the readers' ability to identify positive results, 15 hypertensive patients with known coronary artery disease were also evaluated.

### 2.7. Statistical Analysis

The study was designed to investigate the effect of short-term subcutaneous anti-TNF therapy on the amount of circulating EPC in RA patients. The threshold for significance was set at 0.05. Data were expressed as mean ± standard deviation. Data for matched pairs were analyzed with Wilcoxon signed-rank test. Correlations were evaluated with the Spearman rank correlation test. Statistical analyses were performed using GraphPad Prism version 6.0 (GraphPad software Inc. La Jolla, CA, USA).

The study protocol was approved by the Institutional Review Board of Policlinico Umberto I, Sapienza University of Rome.

## 3. Results

We recruited 17 RA patients (14F:3M, mean age 50.4 + 14.4 years, range 26–68 years) with long-standing disease (mean disease duration 103 ± 104,4 months, range 24–360) who were designed to start a subcutaneous anti-TNF drug. Fourteen were treated with etanercept 50 mg/week/subcutaneously and 3 with adalimumab 40 mg/every other week/subcutaneously. 

At the time of enrollment, all patients were taking glucocorticoids; none of the patients increased steroid dose during the followup. 

Clinical characteristics of RA patients at baseline and after 3 months of anti-TNF treatment are reported in Table 1. After 3 months a significant decrease in DAS28 (versus baseline values) was recorded (*P* = 0.001).

### 3.1. Circulating EPCs

**Figure 2:**
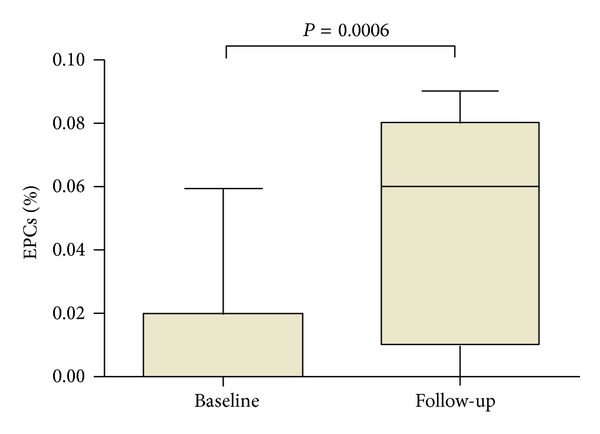
Mean percentage of EPCs before and after 3 months of anti-TNF treatment. EPCs: endothelial progenitor cells.

**Figure 3:**
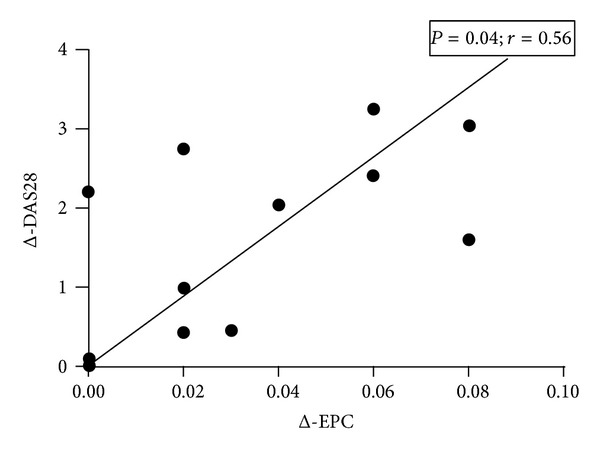
Correlation between mean increase of EPCs and mean decrease of DAS28 after 3 months of anti-TNF treatment. EPCs: endothelial progenitor cells and DAS28: disease activity score 28.

**Figure 4:**
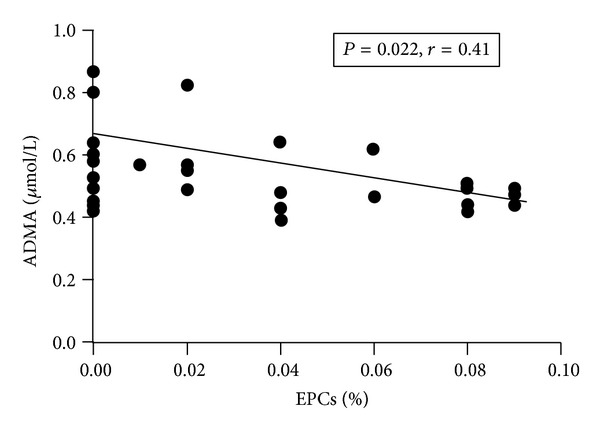
Correlation between mean EPCs and ADMA serum levels in RA patients. EPCs: endothelial progenitor cells, ADMA: asymmetric asymmetric dimethyl arginine.

At baseline, the percentage of circulating EPCs was significantly lower in active RA patients than in healthy subjects (0.01 ± 0.02% versus 0.05 ± 0.03%, *P* = 0.001). At 3 months followup, the number of EPCs was significantly higher compared to basal values (from 0.01 ± 0.02% to 0.05 ± 0.04%, *P* = 0.0006 versus baseline; *P* = n.s.  *versus* healthy subjects) (Figure 2). No significant correlation between EPCs and DAS28 values was detected (*P* = 0.056); However, an inverse correlation between mean increase in EPCs number and mean decrease of DAS28 after 3 months of anti-TNF therapy was observed (*r* = −0.56, *P* = 0.04) (Figure 3). Moreover, EPCs number inversely correlated with ADMA serum levels (*r* = −0.41, *P* = 0.022) (Figure 4). No other correlations between EPCs and clinical characteristic nor FMD values were detected.

### 3.2. ADMA Serum Levels

At baseline, mean ADMA levels were 0.64 ± 0.12 *μ*mol/L. After 3 months of anti-TNF, ADMA serum levels significantly decreased below the values detected before treatment, (0.47 ± 0.04 versus 0.64 ± 0.12 *μ*mol/L, *P* = 0.001). 

### 3.3. Doppler Ultrasound Assessment of Flow Mediated Dilatation

Mean FMD at baseline was 8, 25 ± 0.09% in RA patients, and 4.3 + 0.7% in positive controls (*P* = 0.001). At 3 months followup, a not significant increase of FMD was observed in RA patients (8, 70 ± 0.06%, *P* = 0.49  *versus* baseline). However, even after anti-TNF administration, mean FMD was below the normal value of 10%. 

## 4. Discussion 

The results of our study demonstrate that short-term treatment of RA with TNF inhibitors is associated to an increase in circulating EPCs concurrently to a proportional decrease of disease activity; these findings suggest that therapeutic intervention aimed at suppressing the inflammatory process might also positively affect the “health” of endothelial barrier.

In RA patients, traditional risk factors, genetic predisposition, and inflammatory mechanisms are now recognized to act synergistically in determining the increased risk of subclinical atherosclerosis and consequent CV events [25, 26]. Systemic inflammation is responsible for a proatherogenic profile characterized by oxidative stress, lipid abnormalities, insulin resistance, hypercoagulable state, and upregulation of proatherogenic inflammatory leucocytes [25] each contributing to endothelial injury. Healthy endothelium represents the main regulator of vascular tone, inflammation, and remodeling; consequently, a loss of its function initiates the atherosclerotic process which ultimately leads to the development of the plaque. Different cardiovascular risk factors act on endothelial cells inducing senescence and apoptosis, thus, determining endothelial dysfunction [27]. Growing evidence suggests that EPCs circulating in peripheral blood play a crucial role in endothelium repair. In RA patients, deficiency of circulating EPCs number and functions has been proven [20, 21, 28, 29]. An *in vitro* study demonstrated that endothelial progenitor cells obtained from RA patients showed impaired migratory response to vascular endothelial growth-factor (VEGF) and adhesive properties to mature endothelial cells after stimulation with TNF, when compared to cultured cells from healthy subjects [29]. Other *in vitro* data demonstrated that TNF-alpha negatively affected proliferative, migratory, and adhesive capacity of human EPCs [30]. After TNF-inhibitor administration, a significant increase in adhesion property of EPCs was detected [28].

Concerning the number of circulating EPCs, a reduction of peripheral blood EPCs was described in RA. In their paper, Herbrig et al. aimed at evaluating, *ex vivo* and *in vitro,* number and function of endothelial progenitors in 13 patients with impaired endothelial function; all patients were treated with methotrexate, and 6 out of 13 were also taking anti-TNF drugs [29]. The authors suggested two hypotheses explaining the alteration in EPCs number and function: the inflammatory disease itself and the effect of methotrexate administration [29]. When comparing the frequency of circulating EPCs in patients with high or low disease activity, Grisar et al. [31] observed a significant difference between subjects with active disease and those with low disease activity or in remission who showed EPCs levels comparable to healthy subjects. Differently from the population studied by Herbrig et al. [29], we enrolled long-standing RA patients with moderate-high disease activity (DAS28 ≥ 3.2) nonresponders to standard Disease Modifying Anti-Rheumatic Drugs (DMARDs) and eligible for anti-TNF therapy; in this population we detected a reduced number of circulating EPCs compared to healthy subjects not correlating with disease activity. The homogeneity of our population did not allow any stratification based on disease activity status. In another population of moderately active RA patients, no correlation between circulating EPCs and disease activity was found [20].

Contrary to most published data on endothelial precursors in RA, few studies demonstrated a higher or similar number of circulating EPCs in RA patients compared to patients with other systemic autoimmune diseases or healthy subjects [32–34]. However, different surface markers and techniques were used to characterise endothelial precursor cells. Endothelial precursors represent an extremely rare population among peripheral blood mononuclear cells, and this scarcity contributes to the difficulty in cell isolation and definition [35, 36]. An additional way to define EPCs is to quantify their ability to proliferate by colony forming unit (CFU) assay. With this method, a depletion of peripheral endothelial progenitors was confirmed [20, 31, 34].

Interestingly, besides a decrease in circulating number, rheumatoid synovia seems to be enriched with EPCs suggesting a role for these precursors in local vasculogenesis [37]. Migration of endothelial precursor cells recruited from the peripheral blood through *α*4*β*1 integrin/vascular cell adhesion molecule (VCAM)-1 [38] might explain the depletion in peripheral blood which compromises the endothelial renewal, thus, beginning the atherosclerotic process.

Serum levels of proinflammatory cytokines, such as IL6, showed an inverse correlation with the number of circulating EPCs in RA patients [29]. Recently, in other condition characterized by endothelial impairment, even TNF showed an inverse correlation with the number of circulating EPCs [39]. Given the pivotal role of TNF in the pathogenesis of both RA and atherosclerosis [40], we aimed at investigating the effect of TNF inhibition on markers of endothelial function. A first observation of TNF effect on endothelial precursors in RA patients comes from an *in vitro* study in which the cytokine was demonstrated to impair the CFU formation activity of EPCs, while the addition of TNF-inhibitor infliximab to cultured cells reversed this effect [20]. Moreover, other *in vitro* data showed that TNF was able to stimulate expression of fractalkine on EPC surface, which determines progenitor cell killing by natural killer cells [41]. Further evidence of TNF-mediated effect on the number of circulating EPCs was provided by Ablin et al. [28] who demonstrated *ex vivo* a positive influence of infliximab administration; the authors investigated the effect of a single dose of the anti-TNF drug in 14 RA patients who were already treated with infliximab and methotrexate and observed a significant increase of EPC number and adhesive function 14 days after drug infusion. The improvement of endothelial precursors after treatment was related to a statistically, even if not clinically, significant decrease in DAS28 score (from 5.1 ± 1.4 to 4.2 ± 1.1) [28]. A drug-mediated effect on EPCs number or an indirect effect, working through a reduction of disease activity can be hypothesized; however, it should be considered that patients evaluated in the study were still moderately active after a single infusion of infliximab [28]. One week treatment with intermediate doses of glucocorticoids was also associated to significant reduction of TNF levels and increase of EPC numbers [20].

To the best of our knowledge, the present study is the first one specifically designed to investigate short-term effect of repeated subcutaneous administration of TNF-inhibitor on EPCs. Differently from a previous study investigating the potential effect of TNF inhibition, in our work we enrolled only RA patients who were naive to any biological drugs. We decided to select patients at their first course of anti-TNF drug in order to minimize potential confounding effect of circulating drugs. As expected, in our RA patients, 3 months treatment with TNF blockers significantly decreased disease activity. As previously reported by others [28], parallel to a significant reduction in DAS28 score our patients showed a significant increase in percentage of circulating EPCs which was inversely correlated with the extent of disease activity reduction. Whether the effect of anti-TNF on EPCs increase is related to drug itself rather than indirectly mediated by the reduction of disease activity can be arguable. Normal number of progenitors previously detected in patients with low disease activity [31] is in line with indications of reduced cardiovascular risk among RA patients effectively treated with methotrexate [42]. However, Methotrexate has demonstrated a proapoptotic effect on cultured endothelial precursors which could at least partially contribute to the decrease of circulating EPCs seen in RA patients irrespective of disease activity status [29]. Contrary to the study by Ablin et al. at the time of first evaluation, all our patients were anti-TNF naive; however, similarly to this previous study, 10/17 (58,8%) were already treated with MTX so we cannot definitively exclude a contribution of this drug to the decrease of baseline EPCs.

In 2007 an inverse correlation between EPCs and ADMA serum has been demonstrated [21]. ADMA is an endogenous inhibitor of NO synthase [43] coming into the limelight as a biomarker of endothelial function. Elevated ADMA serum levels have been described in many conditions associated to increased cardiovascular risk, including long-standing and early RA [21, 44, 45]. Moreover, after effective RA treatment both with standard DAMRDs and biological drugs, decreased ADMA serum levels were observed [44, 45]. In the present study, we confirmed a significant reduction in ADMA levels after 3 months of treatment with TNF-inhibitors. This is in line with previous observations that disclosed that anti-TNF blockade led to a decrease of the levels of endothelial cell activation biomarkers in patients undergoing anti-TNF-*α* therapy because of severe disease refractory to conventional therapy [46]. This result, together with the evidence of increased EPCs, further suggests the ability of anti-TNF to reverse the effect of chronic low-grade inflammation on endothelial biomarkers in RA patients. FMD is considered as a noninvasive, standardized method to investigate endothelial function. However, this ultrasonographic technique is limited by operator dependence, and it is related to the environmental conditions in which it is performed [27]. This might explain the reason for nonsignificant improvement of FMD recorded in our patients even if we observed a significant improvement of biomarkers of endothelial function. Even if we detected a slight increase in FMD, the relative small cohort size may partially account for not reaching a statistically significant value.

This small prospective study was designed to evaluate the effect of anti-TNF treatment of EPCs in RA patients. The major shortcoming of this pilot study is the small size of the cohort evaluated and the relatively short followup. Further assessment of endothelial biomarkers on a wider and heterogeneous RA population and longer followup would confirm the result of our study and allow stratifying patients for cardiovascular risk.

In conclusion, our results enhance the current knowledge on the impairment of endothelial biomarkers in RA, as evaluated by EPCs and ADMA. An effective treatment with anti-TNF agents, aimed at reducing disease activity, seems to contribute to the improvement of endothelial barrier function. Such observation suggests a possible role of these drugs in reducing atherosclerotic damage by controlling inflammation.

## Figures and Tables

**Table 1:** 

F/M	14/3
Age—yrs	
Mean ± SD (range)	50.4 ± 14.4 (26–68)
Disease duration—months	
Mean ± SD (range)	103.0 ± 104.4 (24–360)
DAS28 baseline	
Mean ± SD (range)	5.2 ± 1.1 (4.27–7.78)
DAS28 followup	
Mean ± SD (range)	3.3 ± 1.3 (0.56–4.03)*
Glucocorticoid dose (mg) baseline	
Mean ± SD (range)	7.5 ± 5 (5–20)**
Glucocorticoid dose (mg) followup	
Mean ± SD (range)	5.2 ± 0.6 (5–7.5)**
Methotrexate	10/17
Leflunomide	3/17
Sulfasalazine	3/17
Adalimumab	3/17
Etanercept	14/17

DAS28: disease activity score 28.

*P = 0.001 versus baseline value, **prednisone-equivalent.
